# Klf5a in Endoderm Promotes Pharyngeal Cartilage Morphogenesis

**DOI:** 10.3390/ijms262211044

**Published:** 2025-11-14

**Authors:** Wanqiu Li, Zeyao Zhu, Ou Sha, Xia Wang

**Affiliations:** 1 School of Basic Medical Sciences, Shenzhen University Medical School, Shenzhen 518055, China; 2060243055@email.szu.edu.cn; 2 Institute of Advanced Biotechnology, School of Medicine, Southern University of Science and Technology, Shenzhen 518055, China; zhuzy@sustech.edu.cn; 3 Institute of Homeostatic Medicine, School of Medicine, Southern University of Science and Technology, Shenzhen 518055, China; 4 School of Medicine, The Chinese University of Hong Kong (Shenzhen), Shenzhen 518172, China

**Keywords:** zebrafish, craniofacial biology, cell signaling, cell differentiation, craniofacial anomalies, developmental biology

## Abstract

Pharyngeal cartilage, derived from neural crest cells (NCCs), undergoes complex morphogenesis driven by signals from the pharyngeal endoderm. However, the molecular mechanisms governing NCC proliferation and differentiation in response to endoderm-derived signals remain poorly understood. Here, we investigate the role of *klf5a*, a zinc-finger transcription factor expressed in pharyngeal endodermal pouches, in zebrafish pharyngeal cartilage development. Knockdown of *klf5a* using morpholinos minimally affected cranial NCC specification and migration but significantly impaired their proliferation and differentiation in the pharyngeal region. Notably, *klf5a* deficiency reduced expression of *fgfbp2b*, a modulator of FGF signaling, in the pharyngeal endoderm. Co-injection of *klf5a* mRNA rescued the cartilage defects, but injection of *fgfbp2b* mRNA alone failed to restore normal cartilage morphogenesis, suggesting that *fgfbp2b* is not the sole mediator of *klf5a*’s effects. These findings indicate that *klf5a* regulates endodermal signaling to direct NCC-derived pharyngeal cartilage formation, likely through multiple downstream targets including *fgfbp2b*. This study provides insights into the complex molecular network underlying craniofacial development and highlights potential therapeutic targets for craniofacial disorders.

## 1. Introduction

Craniofacial malformations encompass a diverse group of developmental defects affecting the skull and face, often leading to breathing and feeding difficulties, and in severe cases, even mortality [1]. Mandibular hypoplasia is a hallmark of many congenital craniofacial disorders, with the mandible being the only mobile bone in the craniofacial skeleton. Extensive research has elucidated the molecular mechanisms underlying mandibular development, highlighting the crucial role of transcription factors in orchestrating this intricate process.

During vertebrate craniofacial development, the pharyngeal endoderm, adjacent to neural crest cells (NCCs), acts as a critical signaling center that guides NCCs differentiation into cartilage. The absence of endoderm, observed in the *sox32* (*casanova*, *cas*) mutant, leads to hypoplastic cartilage, underscoring the vital role of the endoderm in cartilage development [2]. Similarly, in *van gogh* mutants, defective pharyngeal pouch formation disrupts pharyngeal arch segmentation, despite normal initial NCC migration, highlighting the importance of pharyngeal pouches as signaling hubs for NCC differentiation [3]. This evidence emphasizes the significance of pharyngeal pouches as signaling centers that direct the differentiation of pharyngeal NCCs into cartilage. However, the precise molecular mechanisms by which the endoderm regulates NCC development remain poorly understood.

Several transcription factors essential for chondrogenesis. For instance, RUNX1, primarily known for its role in hematopoiesis, also regulates cartilage formation during bone development and repair [4]. *Sox9* is indispensable for chondrocyte differentiation across all stages of growth plate development, and its loss leads to severe mandibular defects and impaired chondrogenesis [5]. Other transcription factors, including *Runx2*, *Twist1*, *AP-2α*, and *pax1a*, interact with signaling pathways such as Wnt, Bmp, Fgf and EphrinB2a to regulate target gene expression during chondrogenesis [6,7,8,9].

Krüppel-like factor 5 (KLF5), a zinc-finger transcription regulator, modulates gene expression by binding GC-rich promoters. Initially identified in stem cells, KLF5 regulates cell proliferation and differentiation through pathways such as WNT and HIPPO signaling [10,11]. In cartilage, KLF5 promotes degradation via MMP9 and influences proliferation and differentiation in other contexts by regulating FGFBP [12,13]. Fibroblast growth factor binding proteins (FGFBPs) enhance the bioactivity of paracrine fibroblast growth factors (FGFs) by facilitating their interaction with cell surface receptors [14]. FGF signaling is critical for endodermal regulation of NCC development, and craniofacial morphogenesis [15], with *fgf3*, *fgf8*, *fgf4*, *fgf17*, and *fgf24* essential for pharyngeal pouch formation, and *Fgfr1–3* inactivation causing chondrodysplasia [16,17,18]. Additionally, *fgfr4* and other factors like *nkx2.3* and *vwa1* regulate pharyngeal cartilage development via FGF signaling [19].

In this study, we investigate the role of *klf5a*, a zebrafish ortholog of KLF5, in pharyngeal cartilage development. By examining its expression and function, we aim to elucidate the molecular mechanisms by which the pharyngeal endoderm regulates NCC differentiation during mandibular morphogenesis.

## 2. Results

### 2.1. klf5a Is Specifically Expressed in Pharyngeal Endodermal Pouches

**Figure 1:**
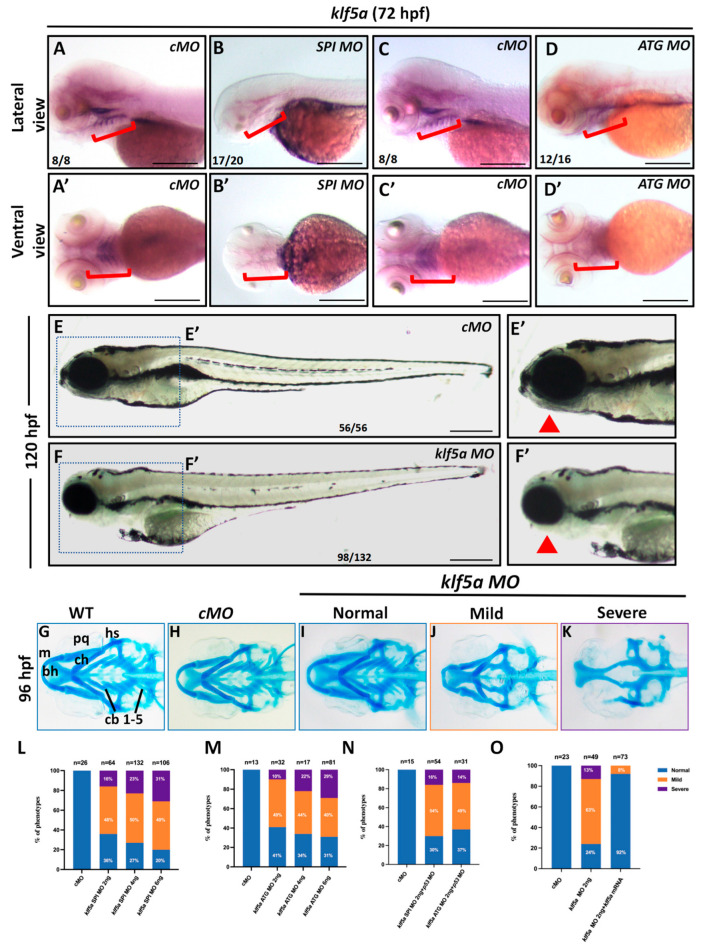
Knockdown of *klf5a* impairs pharyngeal cartilage development in zebrafish. (**A**–**D**) Whole-mount in situ hybridization (WISH) showing *klf5a* mRNA expression in zebrafish embryos at 48 h post-fertilization (hpf). (**A**,**A′**) Control morpholino (cMO)-injected embryos display robust *klf5a* expression in the pharyngeal region (lateral and ventral views, respectively). (**B**,**B′**) Embryos injected with *klf5a* splice-blocking morpholino (SPI MO) show diminished *klf5a* expression. (**C**,**C′**) cMO-injected embryos, as in (**A**,**A′**). (**D**,**D′**) Embryos injected with *klf5a* translation-blocking (ATG) MO exhibit diminished *klf5a* expression. (**E**,**F**) Lateral views of 120 hpf embryos injected with cMO (**E**,**E′**) or *klf5a* SPI MO (**F**,**F′**), showing mandibular hypoplasia and pericardial edema in morphants. (**G**–**K**) Alcian blue staining of cranial cartilage at 96 hpf in wild-type (WT) (**G**), cMO-injected (**H**), and *klf5a* MO-injected embryos (**I**–**K**), revealing absent or reduced cartilage in morphants. (**L**) Quantification of mandibular cartilage phenotype in embryos injected with increasing doses of *klf5a* SPI MO (chi-square test, *p* < 0.0001). (**M**) Quantification of mandibular cartilage phenotype in embryos injected with increasing doses of *klf5a* ATG MO (chi-square test, *p* < 0.0001). (**N**) Quantification of cartilage defects in embryos co-injected with *klf5a* SPI MOs and *p53* MO to rule out off-target effects (chi-square test, *p* < 0.0001). (**O**) Rescue experiments showing restored cartilage formation in *klf5a* MO-injected embryos co-injected with *klf5a* mRNA (chi-square test, *p* < 0.0001). Cartilage structures: m, Meckel′s cartilage; bh, basihyal; ch, ceratohyal; pq, palatoquadrate; hs, hyosymplectic; cb, ceratobranchial. The red brackets (in **A**–**D**, **A′**–**D′**) indicate the domain expression of klf5 mRNA in pharyngeal arches. The red trianges indicate the lower jaw. Scale bar: 50 μm.

**Figure A1:**
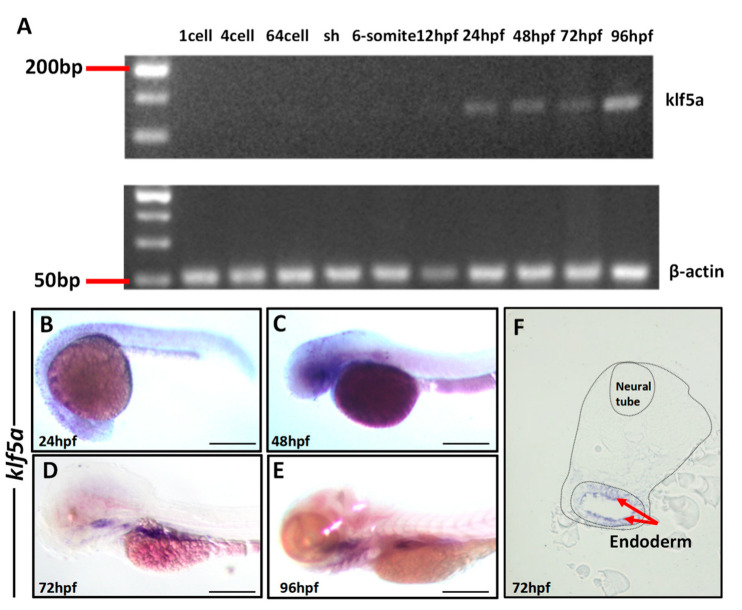
Spatiotemporal Expression of *klf5a* in Zebrafish Embryos. (**A**) Semi-quantitative RT-PCR showing *klf5a* mRNA expression from 1 cell stage to 96 h post-fertilization (hpf). (**B**–**E**) Whole-mount in situ hybridization (WISH) of *klf5a* expression: (**B**) ubiquitous expression at 24 hpf; (**C**) head-enriched expression at 48 hpf; (**D**) specific expression in the pharyngeal region at 72 hpf; (**E**) increased pharyngeal expression at 96 hpf. (**F**) Histological section at 72 hpf showing *klf5a* expression localized to pharyngeal pouch endoderm. The red signal indicates the development of endoderm. Scale bar: 50 μm.

To investigate the role of *klf5a* in zebrafish craniofacial development, we first characterized its spatiotemporal expression during embryogenesis using semi-quantitative reverse transcription PCR (RT-PCR) and whole-mount in situ hybridization (WISH). RT-PCR analysis detected *klf5a* mRNA as early as 24 h post-fertilization (hpf), with expression persisting through 96 hpf, a critical period for pharyngeal cartilage development (Appendix A, Figure A1A). WISH revealed ubiquitous *klf5a* expression throughout the embryo at 24 hpf (Figure A1B), which became progressively restricted to the head region by 48 hpf (Figure A1C). By 72–96 hpf, *klf5a* expression was significantly upregulated in the pharyngeal region, particularly in the endodermal pouches (Figure 1A,C and Figure A1A,D,E). To confirm the precise localization of *klf5a*, we performed histological sectioning of WISH stained embryos at 72 hpf, which demonstrated specific *klf5a* expression in the endodermal cells lining the pharyngeal pouches (Figure A1F). These results indicate that *klf5a* is dynamically expressed in the pharyngeal endoderm during stages critical for NCC-derived cartilage morphogenesis, suggesting a potential role in regulating this process.

### 2.2. klf5a Knockdown Causes Severe Pharyngeal Cartilage Defects

**Figure A2:**
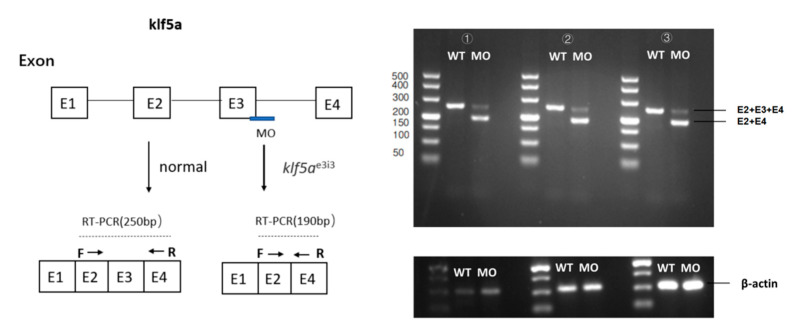
Validation of *klf5a* Knockdown and Downstream Effects. RT-PCR of cDNA from control morpholino (cMO)- and *klf5a* splice-blocking (SPI) MO-injected embryos, showing exon 3 skipping in *klf5a* morphants.

To elucidate the function of *klf5a* in pharyngeal cartilage development, we knocked down its expression using morpholinos (MOs) targeting *klf5a* (Figure A2). Morpholinos were injected into wild-type zebrafish embryos at the 1-cell stage, and knockdown efficacy was validated by WISH, which showed a significant reduction in *klf5a* expression in the pharyngeal region of *klf5a* MO-injected embryos compared to those injected with a control morpholino (cMO) (Figure 1B,D).

Phenotypic analysis revealed that *klf5a* morphants exhibited severe developmental abnormalities, including reduced body length, smaller head size, pericardial edema, and pronounced mandibular hypoplasia compared to cMO-injected embryos (Figure 1E,F). To assess cartilage development, we performed Alcian blue staining at 96 hpf, which revealed a defective cartilage structures in the pharyngeal region of most *klf5a* morphants, in contrast to the well-defined cartilage elements in wild-type and cMO embryos (Figure 1G–K). We categorized the cartilage phenotypes into three types: normal (intact cartilages), mild (absence of hyoid and branchial cartilages), and severe (near-complete loss of all cranial cartilages). The incidence of mild and severe phenotypes increased dose-dependently with higher *klf5a* MO concentrations (Figure 1L,M), confirming a specific effect of *klf5a* knockdown on cartilage morphogenesis.

To exclude off-target effects, we co-injected *klf5a* MO with a *p53* MO to suppress potential morpholino-induced apoptosis. Cartilage defects persisted in co-injected embryos, indicating that the phenotype was specific to *klf5a* knockdown (Figure 1N). To further validate specificity, we performed rescue experiments by co-injecting *klf5a* mRNA with *klf5a* MO. This significantly restored cartilage formation, with 92% embryos exhibiting near-normal pharyngeal cartilage morphology (Figure 1O). These findings collectively demonstrate that *klf5a* is essential for the proper formation of NCC-derived pharyngeal cartilage in zebrafish.

### 2.3. klf5a Deficiency Impairs NCC Proliferation and Differentiation but Not Specification or Migration

**Figure 2:**
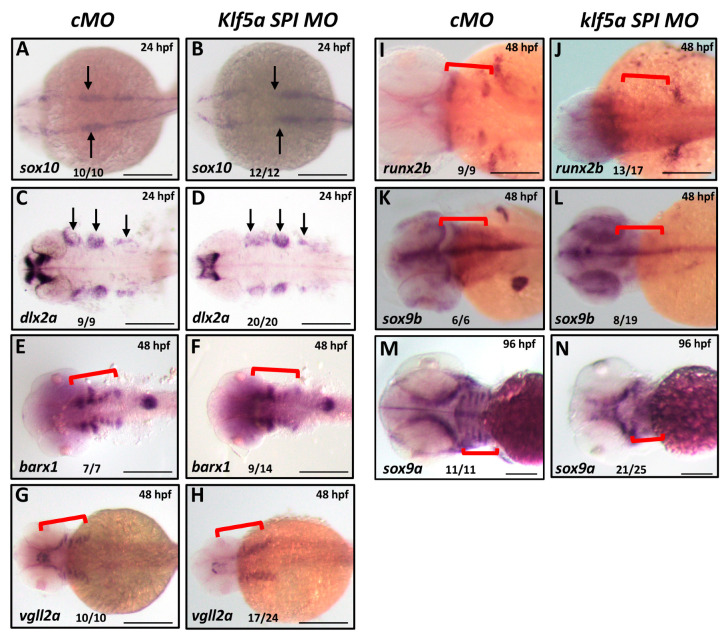
*klf5a* knockdown disrupts NCC differentiation but not specification or migration. Whole-mount in situ hybridization (WISH) assessing neural crest cell (NCC) markers in control morpholino (cMO)- and *klf5a* SPI MO-injected zebrafish embryos. (**A**,**B**) *sox10* expression at 24 hpf, showing normal NCC specification in cMO (**A**) and *klf5a* SPI MO (**B**) embryos. (**C**,**D**) *dlx2a* expression at 24 hpf, indicating intact NCC migration in both groups. (**E**,**F**) *barx1* expression at 48 hpf, revealing reduced NCC aggregation in *klf5a* SPI MO embryos (**F**) compared to cMO (**E**). (**G**,**H**) *vgll2a* expression at 48 hpf, showing decreased NCC aggregation in *klf5a* SPI MO embryos (**H**) compared to cMO (**G**). (**I**,**J**) *runx2b* expression at 48 hpf, indicating impaired chondrocyte differentiation in *klf5a* SPI MO embryos (**J**) compared to cMO (**I**). (**K**,**L**) *sox9b* expression at 48 hpf, reduced in *klf5a* SPI MO embryos (**L**) compared to cMO (**K**). (**M**,**N**) *sox9a* expression at 48 hpf, reduced in *klf5a* SPI MO embryos (**N**) compared to cMO (**M**). The black arrows and red brackets indicate the domain development of neural crest cells. Scale bar: 50 μm.

To determine how *klf5a* regulates NCC development, we investigated its effects on NCC specification, migration, aggregation, and differentiation using WISH with established markers [20]. At 24 hpf, expression of *sox10*, a marker of NCC specification [21], and *dlx2a*, a marker of NCC migration [22], was comparable between *klf5a* morphants and cMO-injected embryos (Figure 2A–D). These results suggest that *klf5a* is not required for the initial specification or migratory phases of cranial NCCs.

We next examined NCC aggregation in the pharyngeal region at 48 hpf using *barx1* [23] and *vgll2a*, markers of NCC condensation prior to chondrogenesis. In *klf5a* morphants, *barx1* and *vgll2a* expression was significantly reduced in the pharyngeal arches compared to controls, indicating impaired NCC aggregation (Figure 2E–H). To assess differentiation, we analyzed the expression of *runx2b* and *sox9a/b*, key markers of chondrocyte differentiation [24]. In *klf5a* morphants, expression of these markers was markedly decreased at 48 and 96 hpf (Figure 2I–N), suggesting defective differentiation of NCCs into chondrocytes.

These findings indicate that *klf5a* is dispensable for NCC specification and migration but plays a critical role in regulating NCC aggregation and differentiation into cartilage in the pharyngeal region.

### 2.4. klf5a Knockdown Does Not Disrupt Pharyngeal Pouch Formation or Endodermal Differentiation

**Figure A3:**
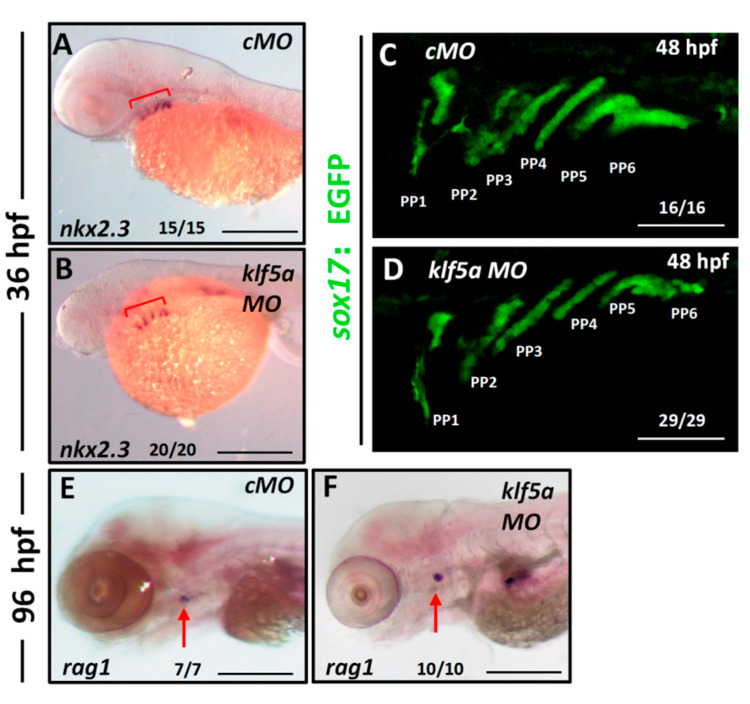
*klf5a* knockdown does not affect pharyngeal pouch formation or endodermal differentiation. (**A**,**B**) Whole-mount in situ hybridization (WISH) showing *nkx2*.3 mRNA expression at 36 hpf in control morpholino (cMO)-injected (**A**) and *klf5a* SPI MO-injected (**B**) embryos, indicating normal pharyngeal pouch segmentation. (**C**,**D**) Immunofluorescence of *sox17*:GFP transgenic embryos at 48 hpf, revealing normal formation of six pharyngeal pouch pairs in cMO (**C**) and *klf5a* SPI MO (**D**) embryos. (**E**,**F**) WISH for *rag1* mRNA at 120 hpf, showing intact thymus differentiation in cMO (**E**) and klf5a SPI MO (**F**) embryos. The red signal indicates the development of pharyngeal pouch. The red arrow indicates the development of thymus. Scale bar: 50 μm.

Given the critical role of the pharyngeal endoderm in regulating NCC development [25,26], we investigated whether *klf5a* knockdown affects pharyngeal pouch formation or endodermal differentiation. We performed WISH for *nkx2.3*, an endodermal marker expressed in pharyngeal pouches [27]. At 48 hpf, *nkx2.3* expression was indistinguishable between *klf5a* morphants and cMO-injected embryos, indicating normal pouch segmentation (Figure A3A,B). To further confirm pouch morphology, we analyzed *sox17*:GFP transgenic embryos, which label the endoderm. In *klf5a* morphants, six pairs of pharyngeal pouches formed normally, with no detectable morphological defects compared to controls (Figure A3C,D).

We also examined endodermal differentiation by analyzing *rag1* [28], a marker of thymus development, at 96 hpf. The expression of *rag1* was unaffected in *klf5a* morphants, suggesting that *klf5a* knockdown does not impair endodermal differentiation into thymus tissue (Figure A3E,F). These results collectively demonstrate that *klf5a* is not required for pharyngeal pouch formation or endodermal differentiation, suggesting that its role in cartilage development is specific to regulating NCC behavior rather than endodermal development.

### 2.5. klf5a Promotes NCC Proliferation in the Pharyngeal Region

**Figure 3:**
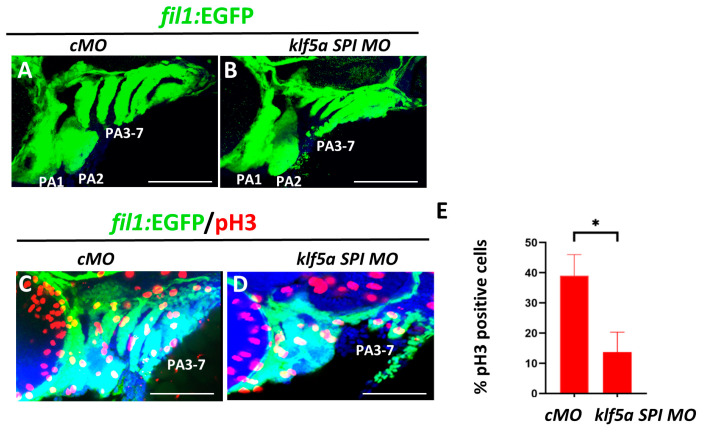
*klf5a* knockdown reduces NCC proliferation in the pharyngeal arches. Immunofluorescence analysis of *fli1*:GFP transgenic zebrafish embryos at 48 hpf. (**A**,**B**) Whole-embryo imaging with DAPI and GFP staining, showing normal pharyngeal arch development in control morpholino (cMO)-injected embryos (**A**) and smaller, disorganized arches in *klf5a* SPI MO-injected embryos (**B**). (**C**,**D**) Phosphorylated histone H3 (pH3) immunofluorescence, indicating reduced proliferation in the pharyngeal arches of *klf5a* SPI MO embryos (**D**) compared to cMO (**C**). (**E**) Quantification of pH3-positive cells in the pharyngeal arches, showing a significant decrease in *klf5a* SPI MO embryos (* *p* < 0.01, Student′s *t*-test). *n* = 19 for cMOs and *n* = 22 for klf5a MO. Scale bar: 50 μm.

To further explore the cellular mechanisms underlying the role of klf5a in NCC regulation, we examined pharyngeal arch development and NCC dynamics using fli1:GFP transgenic zebrafish, which label NCCs in the pharyngeal arches. Immunofluorescence at 48 hpf revealed that klf5a morphants exhibited significantly smaller and disorganized pharyngeal arches compared to cMO-injected controls, which displayed seven well-formed arch pairs (Figure 3A,B).

**Figure A4:**
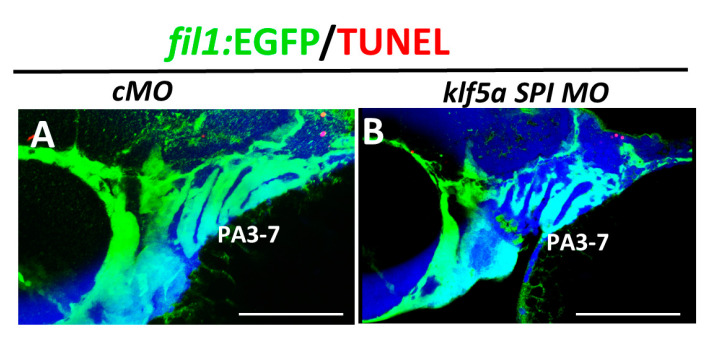
(**A**,**B**) TUNEL assay detecting apoptosis in cMO (**B**) and *klf5a* SPI MO (**A**) embryos, with minimal apoptotic signals in the pharyngeal region of both groups.

We hypothesized that klf5a knockdown might affect NCC proliferation or survival. To test this, we performed TUNEL assays to detect apoptosis in fli1:GFP embryos at 48 hpf. Minimal apoptotic signals were observed in both klf5a morphants and controls, with no specific increase in apoptosis in the pharyngeal region of morphants (Figure A4A,B). In contrast, immunofluorescence for phosphorylated histone H3 (pH3), a marker of cell proliferation, revealed a significant reduction in pH3-positive cells in the pharyngeal arches of klf5a morphants compared to controls (Figure 3D). Quantitative analysis confirmed a statistically significant decrease in NCC proliferation in the pharyngeal region (Figure 3E).

These findings suggest that klf5a promotes NCC proliferation in the pharyngeal arches but does not affect their survival, providing a mechanistic basis for the observed cartilage defects in klf5a morphants.

### 2.6. Klf5a Regulates Pharyngeal Cartilage Development via fgfbp2b

**Figure 4:**
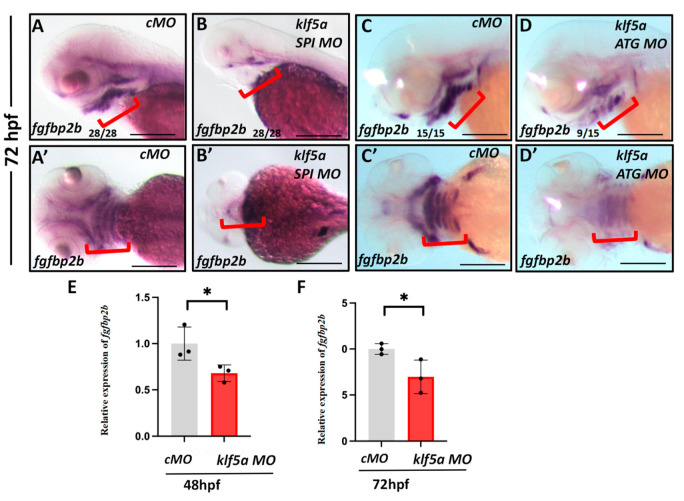
*klf5a* regulates *fgfbp2b* expression in the pharyngeal endoderm. (**A**–**D**) Whole-mount in situ hybridization (WISH) showing *fgfbp2b* mRNA expression at 72 hpf in zebrafish embryos. (**A**,**A′**) Control morpholino (cMO)-injected embryos display robust *fgfbp2b* expression in the pharyngeal endoderm (lateral and dorsal views, respectively). (**B**,**B′**) *klf5a* splice-blocking (SPI) MO-injected embryos show reduced *fgfbp2b* expression (lateral and dorsal views, respectively). (**C**,**C′**) cMO-injected embryos, as in (**A**). (**D**,**D′**) *klf5a* translation-blocking (ATG) MO-injected embryos exhibit reduced *fgfbp2b* expression (lateral and dorsal views, respectively). (**E**,**F**) Quantitative RT-PCR (qRT-PCR) analysis of *fgfbp2b* expression at 48 hpf (**E**) and 72 hpf (**F**) in cMO and *klf5a SPI* MO embryos, confirming significant downregulation in morphants (* *p* < 0.01, Student′s *t*-test). The red brackets indicate the domain expression of *fgfbp2b* during pharyngeal development. Scale bar: 50 μm.

To identify the downstream targets of *klf5a*, we performed RNA sequencing on *klf5a* morphant embryos at 48 hpf. Transcriptome analysis revealed a significant downregulation of *fgfbp2b*, a gene encoding a fibroblast growth factor binding protein, in the pharyngeal endoderm of *klf5a* morphants. WISH confirmed decreased *fgfbp2b* expression specifically in the pharyngeal pouches of *klf5a* morphants at 48 hpf (Figure 4A–D). qRT-PCR validated a significant reduction in *fgfbp2b* mRNA levels (Figure 4E,F). Given the role of FGF-BP in enhancing FGF signaling [29], which is critical for endodermal regulation of NCC proliferation and differentiation [14], we hypothesized that *klf5a* regulates cartilage development through *fgfbp2b*.

**Figure 5:**
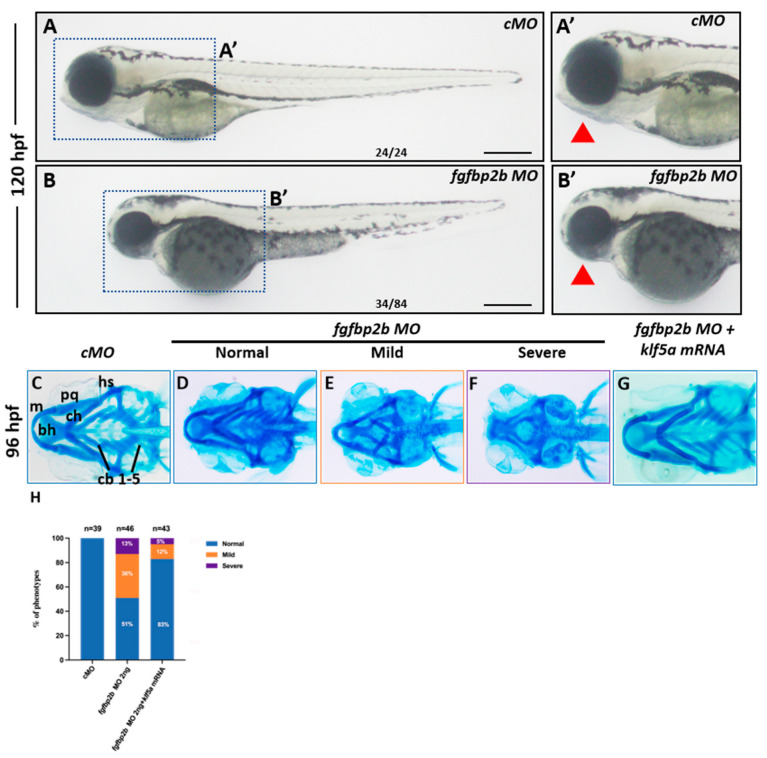
*fgfbp2b* contributes, to but does not fully mediate, *klf5a*-dependent pharyngeal cartilage development. (**A**,**A′**,**B**,**B′**) Lateral views of zebrafish embryos at 120 hpf, showing normal morphology in control morpholino (cMO)-injected embryos (**A**,**A′**) and mandibular hypoplasia in *fgfbp2b* MO-injected embryos (**B**,**B′**). (**C**–**F**) Alcian blue staining of cranial cartilage at 96 hpf in cMO (**C**) and *fgfbp2b* MO embryos (**D**–**F**), revealing mild to severe cartilage defects in morphants. (**G**) Representative images of Alcian blue-stained embryos from the rescue experiment, showing restored cartilage in *fgfbp2b* MO and *klf5a* mRNA co-injected embryos. (**H**) Quantification of cartilage defects in *fgfbp2b* MO embryos (47% with defects) and *fgfbp2b* MO embryos co-injected with *klf5a* mRNA (83.3% normal), (Chi-square test, *p* < 0.001). Cartilage structures: m, Meckel’s cartilage; bh, basihyal; ch, ceratohyal; pq, palatoquadrate; hs, hyosymplectic; cb, ceratobranchial. The red trianges indicate the lower jaw.

To test this, we knocked down *fgfbp2b* using specific MOs, which resulted in reduced *fgfbp2b* expression and mandibular hypoplasia, phenocopying *klf5a* morphants (Figure 5A,B). Alcian blue staining at 96 hpf revealed mild to severe cartilage defects in *fgfbp2b* morphants, similar to those in *klf5a* morphants (Figure 5C–F,H).

To investigate whether *fgfbp2b* mediates the effects of *klf5a*, we performed rescue experiments by co-injecting *klf5a* MO with *fgfbp2b* mRNA. However, *fgfbp2b* mRNA injection failed to restore cartilage formation in *klf5a* morphants, suggesting that *fgfbp2b* is not the sole mediator of *klf5a*’s effects. In contrast, co-injection of *fgfbp2b* MO with *klf5a* mRNA significantly reduced the incidence of cartilage defects, with many embryos showing restored cartilage formation (Figure 5G,H).

We also examined *runx3* and *sox9b* expression in *fgfbp2b* morphants and found it significantly reduced (Figure A4B). These results suggest that *klf5a* regulates *fgfbp2b* expression in the pharyngeal endoderm, which in turn modulates NCC proliferation and differentiation, thereby promoting pharyngeal cartilage morphogenesis.

## 3. Discussion

The development of vertebrate craniofacial structures, particularly pharyngeal cartilage, relies on a complex signaling network orchestrated by the pharyngeal endoderm to regulate neural crest cell (NCC) differentiation and morphogenesis. In this study, we demonstrate that *klf5a*, a zinc-finger transcription factor specifically expressed in the endodermal pouches of zebrafish, plays a critical role in pharyngeal cartilage formation by modulating *fgfbp2b* expression and likely other downstream targets. Our findings reveal that *klf5a* knockdown does not affect cranial NCC specification or migration but significantly impairs their proliferation and differentiation in the pharyngeal region. These results highlight *klf5a* as a key regulator of later-stage NCC development, specifically in the context of endodermal signaling that directs cartilage morphogenesis.

Previous studies have underscored the conserved role of KLF5 in cartilage development across vertebrates. For instance, Klf5 acts as a downstream effector of Erg1 in articular cartilage, contributing to chondrocyte hypertrophy and degeneration in osteoarthritis [30]. In Klf5 heterozygous (Klf5+/−) mice, skeletal development is altered due to impaired cartilage matrix degradation, leading to delayed ossification and an elongated hypertrophic chondrocyte layer in neonatal growth plates [31]. This phenotype is partly attributed to reduced expression of Mmp9, a matrix metalloproteinase directly regulated by Klf5, which is critical for matrix remodeling and vascularization during endochondral ossification. Similarly, ectopic *klf5a* expression in zebrafish larvae disrupts head cartilage formation, including ceratobranchial, ceratohyal, and Meckel’s cartilage. Our results align with these findings, demonstrating that the important role of *klf5a* knockdown in zebrafish leads to severe mandibular hypoplasia and loss of pharyngeal cartilage structures, likely due to defective NCC proliferation and differentiation. However, unlike in mice, where Klf5 primarily affects matrix degradation, our data suggest that *klf5a* in zebrafish primarily regulates NCC dynamics, indicating species-specific or context-dependent roles for Klf5 in cartilage development.

The pharyngeal endoderm is a well-established signaling center that directs NCC differentiation into cartilage [32]. In zebrafish *sox32* mutants, endoderm ablation results in hypoplastic cartilage, while *van gogh* mutants with defective pharyngeal pouch formation exhibit disrupted arch segmentation [2,33]. Our observation that *klf5a* knockdown does not impair pharyngeal pouch formation or endodermal differentiation, as evidenced by normal *nkx2.3* and *rag1* expression and *sox17*:GFP transgenic analysis, suggests that the role *klf5a* is specific to endodermal signaling rather than pouch morphogenesis. This is consistent with the role of other endodermal signaling molecules, such as *edn1*, which is expressed in the pharyngeal endoderm and signals through *ednrA1* and *ednrA2* receptors in NCCs to promote cartilage formation [34]. Knockdown of *edn1* or its receptors results in cartilage loss similar to that observed in *klf5a* morphants, supporting the hypothesis that *klf5a* regulates endodermal signals critical for NCC development.

Our finding that *klf5a* knockdown downregulates *fgfbp2b* expression in the pharyngeal endoderm provides a mechanistic link to cartilage defects. Fibroblast growth factor binding proteins (FGFBPs) enhance FGF signaling by facilitating ligand-receptor interactions [14,35]. FGF signaling is essential for craniofacial development, with *fgf3*, *fgf8*, *fgf4*, *fgf17*, and *fgf24* driving pharyngeal pouch formation and *fgfr1–3* supporting chondrogenesis [16,36,37]. The reduced *fgfbp2b* expression in *klf5a* morphants likely disrupts FGF signaling, impairing NCC proliferation and differentiation, as evidenced by decreased expression of *barx1*, *vgll2a*, *runx2b*, and *sox9a/b*. However, our rescue experiments demonstrate that *fgfbp2b* mRNA injection does not restore cartilage formation in *klf5a* morphants, indicating that *fgfbp2b* is not the sole mediator of *klf5a*’s effects. The incomplete efficiency of morpholino-mediated *klf5a* knockdown, which may result in residual *klf5a* protein activity, could contribute to this outcome, as *fgfbp2b* overexpression may not fully compensate for the broader regulatory network controlled by *klf5a*. Due to funding constraints, we were unable to perform RNA-seq or ChIP-seq to identify additional *klf5a* targets, but the partial phenocopy of *klf5a* morphants by *fgfbp2b* knockdown and the reduction in *runx3* and *sox9b* expression in *fgfbp2b* morphants support a model where *fgfbp2b* is one of several factors regulated by *klf5a*.

The specificity of *klf5a* on NCC proliferation and differentiation, but not specification or migration, distinguishes it from other transcription factors like *sox9* and *runx2*, which broadly regulate NCC development. This stage-specific role suggests that *klf5a* fine-tunes endodermal signaling during the critical transition from NCC aggregation to chondrocyte differentiation. The absence of apoptosis in *klf5a* morphants further indicates that the cartilage defects result from proliferative and differentiative failures rather than cell loss, reinforcing the importance of *klf5a* in sustaining NCC populations in the pharyngeal arches.

Future studies should explore the direct transcriptional targets of *klf5a* in the pharyngeal endoderm, particularly whether it binds the *fgfbp2b* promoter, and investigate potential interactions with other signaling pathways, such as WNT or BMP, which also regulate craniofacial development [38]. The conserved role of *klf5* across species suggests that insights from zebrafish could inform studies of craniofacial disorders in humans, where FGF signaling disruptions are implicated in conditions like Pierre Robin sequence and Treacher Collins syndrome [19,39,40]. By elucidating the role of *klf5a* in endodermal regulation of NCC-derived cartilage, this study provides a foundation for understanding the molecular basis of craniofacial morphogenesis and identifying therapeutic targets for congenital craniofacial anomalies.

## 4. Materials and Methods

### 4.1. Zebrafish Husbandry and Strains

We used zebrafish (*Danio rerio*) lines: wild type (AB), *Tg(sox17:GFP)*, *Tg(fil1:GFP)*, *Tg(sox10:GFP)*, obtained from China Zebrafish Resource Center-National Aquatic Biological Resource Center (NABRC-CZRC, Shanghai, China). Embryos were collected post-fertilization and maintained at 28.5 °C in the embryo medium (5 mM NaCl, 0.17 mM KCl, 0.33 mM CaCl_2_, 0.33 mM MgSO_4_, and 0.002% methylene blue; all chemicals sourced from Sigma-Aldrich, St. Louis, MO, USA). Embryos were staged by morphology as previously described [41]. All animal experiments complied with the guidelines approved by the Committee on Animal Experimentation of Shenzhen University. Animal work followed the Animal Research: Reporting of In Vivo Experiments 2.0 (ARRIVE 2.0) guidelines.

### 4.2. Morpholino (MO) and Microinjections

We designed two different MO-targeting *klf5a* based on previous reports [42] The ATG MO targets the mRNA translation initiation site, preventing its normal translation, while the Splice MO targets the site between the third intron and the third exon, preventing normal splicing (Figure A2). We injected 2–4 ng of control MO (5′-CCTCTTAC CTCAGTTACAATTTATA-3′) or 2–4 ng of *klf5a* Exon3 splice MO (5′-TTAATTGCGGAACTCTT ACCAGTGT-3′) or 2–4ng of *klf5a* ATG MO (5′-GTAAGAAGCGTAGCGGCCATAAACC-3′) into embryos. All morpholinos were synthesized by Gene Tools, LLC (Philomath, OR, USA).

### 4.3. Whole-Mount In Situ Hybridization

cDNA fragments for *klf5a*, *sox9a*, *sox9b*, *runx3*, *runx2b sox10*, *barx1*, *nkx2.3*, *rag1* and *fgfbp2b* were amplified by RT-PCR with specific primers (Table S2), Digoxigenin-UTP-labeled antisense RNA probes were then synthesized using a Transcription Kit (Roche, Basel, Switzerland, Catalog No. 11175025910) following the manufacturer’s instructions. The probes were purified with a purification kit (Roche, Basel, Switzerland, Catalog No. 940200) and then stored at −80 °C. Whole-mount in situ hybridization was performed as previously described [43].

### 4.4. Quantitative Real-Time PCR

For quantitative real-time PCR (qRT-PCR), total RNA was extracted from individual zebrafish embryos using TRIzol reagent, and the first-strand cDNA was synthesized with GoScript™ Reverse Transcriptase (Promega, Madison, WI, USA, Catalog No. A5002). Applied Biosystems was employed to perform qRT-PCR using SYBR^®^ Green Ex Taq™ dye (Takara Bio, Kusatsu, Shiga, Japan, Catalog No. NJ400). The target genes were amplified with the primers listed in Table S1. Expression levels of the investigated genes were quantified via the 2^−ΔΔCT^ method and normalized to β-actin transcripts. Statistical analysis was carried out with an unpaired *t*-test.

### 4.5. Immunofluorescence and TUNEL Assay

Zebrafish embryos were fixed in 4% paraformaldehyde (Sigma-Aldrich, St. Louis, MO, USA) overnight at 4 °C. Fixed embryos were then washed three times with PBST (PBS + 0.2% Triton-X100) for 5 min each. After being treated with proteinase K (10 μg/mL, Sigma-Aldrich, St. Louis, MO, USA) at room temperature, the embryos were blocked with 10% heat-inactivated goat serum (Sigma-Aldrich, St. Louis, MO, USA) at room temperature for 1 h. The embryos were then stained overnight at 4 °C with the following primary antibodies: anti-GFP (1:500; Abcam, Cambridge, UK) and anti-PH3 (1:500; Cell Signaling Technology, Danvers, MA, USA). Samples were washed 3 times with PBST, and incubated with secondary antibodies (Alexa Fluor, Thermo Fisher Scientific, Waltham, MA, USA). The stained embryos were embedded with 2% low melting agarose and imaged using an Olympus confocal microscope (Olympus, Tokyo, Japan).

For the TUNEL assay, collected embryos were fixed overnight at 4 °C in paraformaldehyde (Sigma-Aldrich, St. Louis, MO, USA), followed by triple washes in PBST and proteinase K-mediated permeabilization. The permeabilized embryos were re-fixed in 4% paraformaldehyde for 30 min at room temperature, washed three times in PBST, and incubated with TUNEL staining according to the manufacturer’s instructions (Roche, Basel, Switzerland, Catalog No. 1215679210).

### 4.6. Alcian Blue Staining

Embryos were fixed, washed, and stained with Alcian blue (Sigma-Aldrich, St. Louis, MO, USA). After staining, embryos were rehydrated and de-stained for imaging.

### 4.7. Rescue Experiments

To perform rescue experiments, we amplified the full length klf5a transcript from WT embryos cDNA (Table S3). The sequences were then cloned into pCS2+ vector (Thermo Fisher Scientific, Waltham, MA, USA). To obtain klf5a mRNA, in vitro transcription was performed using the mMESSAGE mMACHINE SP6 kit (Ambion, Austin, TX, USA, Carlsbad, CA, USA) and polyadenylation was performed using the Poly (A) Tailing Kit The purified klf5a mRNAs (100 pg/embryo) were microinjected into one-cell stage embryos.

### 4.8. Statistical Analysis

The qRT-PCR results were analyzed using a *t*-test with Prism software (GraphPad v9.5.1, San Diego, CA, USA). Immunofluorescence signals were analyzed by ImageJ software 1.53k (National Institutes of Health, Bethesda, MD, USA). To compare the percentage of abnormal embryos across multiple groups, a Chi-square test of independence was used. Statistical significance was determined, with * indicating *p* < 0.05, ** indicating *p* < 0.01, and *** indicating *p* < 0.001.

## Figures and Tables

**Figure A5:**
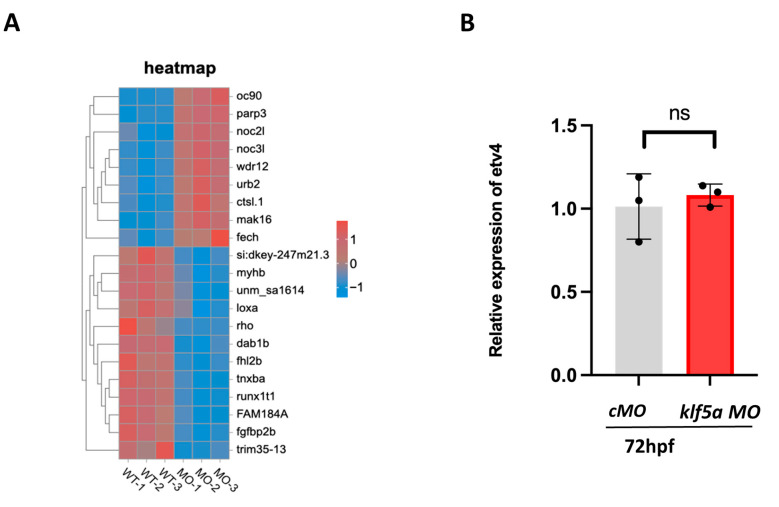
*kl5a* sequencing and analysis. (**A**) Cluster analysis of differentially expressed genes after *klf5a* knockdown (**B**). Quantitative RT-PCR (qRT-PCR) analysis of *etv4* expression at 72 hpf in cMO and *klf5a SPI* MO embryos. The black dots represent the results of the three samples.

**Figure A6:**
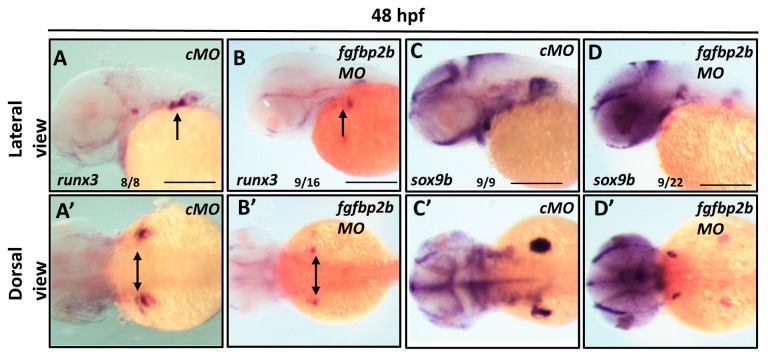
*fgfbp2b* knockdown disrupts chondrogenic marker expression. (**A**,**A′**,**B**,**B′**) Whole-mount in situ hybridization (WISH) showing *runx3* mRNA expression at 48 hpf in control morpholino (cMO)-injected (**A**,**A′**) and *fgfbp2b* MO-injected (**B**,**B′**) zebrafish embryos, indicating reduced expression in morphants. (**C**,**C′**,**D**,**D′**) WISH for *sox9b* at 48 hpf, showing decreased expression in *fgfbp2b* MO embryos (**D**,**D′**) compared to cMO (**C**,**C′**). The arrow indicates the expression of runx3. Scale bar: 50 μm.

## Data Availability

The data used to support the findings of this study are available from the corresponding author upon reasonable request.

## Supplementary Materials

The following supporting information can be downloaded at: https://www.mdpi.com/article/10.3390/ijms262211044/s1.
