# Supplementary material for: Klf5a in Endoderm Promotes Pharyngeal Cartilage Morphogenesis
Source: Int J Mol Sci. 2025 Nov 14;26(22):11044. doi: 10.3390/ijms262211044 (PMC12652733; doi:10.3390/ijms262211044)
Supplement: Supplementary file 1 [file ijms-26-11044-s001.zip › ijms-3905330-supplementary.pdf]

Table S1. The sequences of the primers for RT-qPCR in this study.

Primer	Forward	Reverse
fgfbp2b	ATACTGCTGGAAGAGCCTG	CACTGGTAACCAGTTGTAGTG
etv4	CTTACGAACCTGATGTGCC	CTTCACTGTTGTGCTGTGG
klf5a	ACGTCCCTGATCCAACTGAC	CGTCTTTTCTCGAGGTCTGG
β-actin	CGAGCAGGAGATGGGAACC	CAACGGAAACGCTCATTGC

Table S2. The sequences of the primers for probe in this study.

Primer	Forward	Reverse
klf5a	ACGTCCCTGATCCAACTGAC	CGTCTTTTCTCGAGGTCTGG
sox10	GGTCAGCCACAGCCAATCG	CACGGTCGAGACAGTGTGG
dlx2a	CCTGAAATCCGAATGGTCAATG	GTCTCACCACTCAAGACTGCC
vgll2a	AGGACTCGACGCGGAGCTTCA	AGAAGTGGCCGCAGGGCTTTA
sox9a	GCACATCAGCTACGGTTCCT	GAGAGCGAGTGGAATGCTGT
barx1	GGGCGGATCAGACTTCTCAC	ATACCCGTCTGGTCTGCCTT
runx2b	TGTCGGTGAAGATGAACGACGT	TCAATACGGCCTCCAAACGCC
fgfbp2b	ACACCAAAGCCAAAGACACC	CTGATGAAGTACGCGCAGAC
sox9b	ATGAATCTCCTCCAGCGCGGG	TCAGGGTCTGGACAGCTGTGT
runx3	ATGGCTTCAAACAGCATCTTC	TTAGTACGGCCTCCAGACAGA
nkx2.3	TCGTCGTGTTTTCTCGGAGG	GCGCATTACTCCACGTGTTC
rag1	AGATTCAGGAGGGACTCG	ACGGGTCAGTGACAACAG

Table S3. The sequences of the primers for mRNA in this study.

Primer	Forward	Reverse
klf5a	GAATTCATGGCCGCTACGCTTCTTACCATG	TCTAGACTAATTCTGATGCCTCTTCATATG
